# Hydrogen generation of single alloy Pd/Pt quantum dots over Co_3_O_4_ nanoparticles via the hydrolysis of sodium borohydride at room temperature

**DOI:** 10.1038/s41598-022-21064-z

**Published:** 2022-10-11

**Authors:** Mostafa Farrag, Gomaa A. M. Ali

**Affiliations:** 1 Nanoclusters and Photocatalysis Laboratory, Chemistry Department, Faculty of Science, Assiut University, Assiut, 71516 Egypt; 2 Chemistry Department, Faculty of Science, Al-Azhar University, Assiut, 71524 Egypt

**Keywords:** Chemistry, Energy science and technology, Materials science, Nanoscience and technology

## Abstract

To satisfy global energy demands and decrease the level of atmospheric greenhouse gases, alternative clean energy sources are required. Hydrogen is one of the most promising clean energy sources due to its high chemical energy density and near-zero greenhouse gas emissions. A single alloyed phase of Pd/Pt nanoclusters as quantum dots (QDs) was prepared and loaded over Co_3_O_4_ nanoparticles with a low loading percentage (1 wt.%) for hydrogen generation from the hydrolysis of NaBH_4_ at room temperature. L-glutathione (SG) was used as a capping ligand. It was found that the single alloy catalyst (Pd_0.5_–Pt_0.5_)_n_(SG)_m_/Co_3_O_4_ caused a significant enhancement in hydrogen generation in comparison to the monometallic clusters (Pd_n_(SG)_m_ and Pt_n_(SG)_m_). Moreover, the Pd/Pt alloy showed a positive synergistic effect compared to the physical mixture of Pd and Pt clusters (1:1) over Co_3_O_4_. The QDs alloy and monometallic Pd and Pt clusters exhibited well-dispersed particle size in ~ 1 nm. The (Pd_0.5_–Pt_0.5_)_n_(SG)_m_)/Co_3_O_4_ catalyst offers a high hydrogen generation rate (HGR) of 8333 mL min^− 1^ g^− 1^ at room temperature. The synergistic effect of Pd and Pt atoms in the nanoclusters alloy is the key point beyond this high activity, plus the prepared clusters' unique atomic packing structure and electronic properties. The effect of the NaBH_4_ concentration, catalyst amount, and reaction temperature (25–60 °C) were investigated, where HGR reaches 50 L min^− 1^ g^− 1^ at 60 °C under the same reaction conditions. The prepared catalysts were analyzed by UV–Vis, TGA, HR-TEM, XRD, and N_2_ adsorption/desorption techniques. The charge state of the Pd and Pt in monometallic and alloy nanoclusters is zero, as confirmed by X-ray photoelectron spectroscopy analysis. The catalysts showed high recyclability efficiency for at least five cycles due to the high leaching resistance of the alloy nanoclusters within the Co_3_O_4_ host. The prepared catalysts are highly efficient for energy-based applications.

## Introduction

Bimetallic systems as heterogeneous catalysts have received tremendous scientific and industrial attention due to their excellent catalytic performance compared to monometallic systems^1–3^. The synergistic effect and cooperative interactions between the two metals are the main reason beyond their activity, where the electrons transfer or exchange between the two metals in the bimetallic lattice enhances the catalytic activity of the bimetallic systems^1,2,4^.

Recently, hydrogen as clean energy has been considered a key factor in the increasing depletion of fossil fuels and exacerbating the environmental pollution crisis^5,6^. Hydrogen is universally accepted as a clean energy source due to its relatively high energy density (142 MJ kg^− 1^) in comparison to diesel and gasoline (46 MJ kg^− 1^) and can reduce the amount of greenhouse gas and acid rain when used as fuel^7^. Hydrolysis of sodium borohydride (NaBH_4_) has been reported to be a promising way to generate hydrogen due to its high hydrogen content (10.65 wt.%), low price, and nontoxicity (NaBH_4_ + 2H_2_O → NaBO_2_ + 4H_2_, ΔH =  − 300 kJ mol^− 1^)^6–12^. Metal alloys such as NiB and CoB showed good activity in the hydrolysis of NaBH_4_^9,10,13^, where 5 wt. % CoB/CeO_2_ showed hydrogen generation rate (HGR) of 533 mL g^− 1^ min^− 1^^10^. Patel et al.^14^ reported the effect of electron transfer between metallic Co and B.

Noble metals such as Au, Pd, Ru, and Pt-based catalysts were reported as ideal candidates for hydrogen generation due to their excellent catalytic activity and high stability^11,12,15^. 20 wt.% of Ru (19.9 nm), Pd (12.2 nm), and Pt (3.2 nm) over Co_3_O_4_ showed high catalytic activity in hydrolysis of NaBH_4_ with HGR of 6514, 4713, and 2109 mL g^− 1^ min^− 1^, respectively^11^. Pt/Al_2_O_3_, Rh/Al_2_O_3_, and Pd/Al_2_O_3_ exhibited some catalytic activity in hydrolysis of 15–23 wt.% NaBH_4_ solutions^16^. The bimetallic catalysts showed higher performance in the hydrolysis of NaBH_4_ than the corresponding monometallic^17–23^. Pt-Ru bimetallic system over LiCoO_2_ support exhibited catalytic activity in the hydrolysis of NaBH_4_ more than the monometallic Ru or Pt^20^. The Ru-Co alloys over γ-Al_2_O_3_ showed higher HGR in NaBH_4_ hydrolysis than single monometallic samples^21^. Ni − Pt/CeO_2_ over granular activated carbon catalyst achieved complete and rapid decomposition of hydrazine hydrate to generate H_2_ with a 100% selectivity at moderate temperature in the presence of 1 M NaOH^22^. Moreover, the salisylaldimine-Ni complex has shown 190% increment in NaBH_4_ hydrolysis than pure Ni catalyst with a maximum hydrogen production rate of 2240 ml min^−1^ g^−1^^24^.

To prepare size-selected metal nanoclusters (quantum dots, QDs), a great deal of work should be done; for example, deposition of Pt clusters over single-crystal supports such as MoO_3_ and TiO_2_ under ultrahigh vacuum (UHV) conditions 10^− 9^ mbar^25^. This model of catalysis is very complicated and largely deviated from the real world of catalytic conditions. Monolayer-protected nanoclusters of noble metals have recently gained much attention in the catalysis field due to their unique atomic packing structure and electronic properties^26–38^. In our previous work, platinum nanoclusters protected with l-cysteine and N-acetyl-l-cysteine over TiO_2_ showed high catalytic activity in the oxidation of styrene^26^ and solar degradation of methylene blue^38^, respectively. Pd nanoclusters protected with N-acetyl-l-cysteine exhibited superior catalytic activity in hydrogenation of α, β-unsaturated aldehydes such as cinnamaldehyde^33^. Moreover, size-selected gold clusters (Au_25_(SCH_2_CH_2_Ph)_18_, Au_38_(SCH_2_CH_2_Ph)_24,_ and Au_144_(SCH_2_CH_2_Ph)_60_) over ceria showed high catalytic activity in the oxidation of carbon monoxide at a low temperature^35–37^.

To the best of our knowledge, this is the first time to prepare a single alloyed Pd/Pt quantum dots (QDs) in regime of 1 nm by simple way in a large yield. A very low loading percentage (1 wt.%) from the prepared alloy over Co_3_O_4_ achieved a high hydrogen yield with a hydrogen generation rate (HGR) 8333 mL g^−1^ min^−1^. Additionally, a comparison between the activity of the single alloy and the physical mixing of Pd and Pt nanoclusters was studied, where the alloy exhibited a positive synergistic effect and the physical mixing showed a negative synergistic effect. Where, the physical mixture of palladium and platinum nanoclusters (1:1) was loaded over the Co_3_O_4_ nanoparticles for comparison. 1 wt.% (Pd_0.5_–Pt_0.5_)_n_(SG)_m_/Co_3_O_4_ catalyst showed the best catalytic activity in NaBH_4_ hydrolysis reaction to generate hydrogen gas. However, the physical mixing of Pd and Pt nanoclusters over Co_3_O_4_ showed lower activity than monometallic platinum clusters. The synergistic effect between Pd and Pt atoms in the nanoclusters alloy plays the main role in this activity. As confirmed by HR-TEM, the particle size of palladium, platinum, and single alloy nanoclusters is around 1 nm. The stoichiometric ratio between the metallic and organic parts in the three prepared nanoclusters was determined by TG analysis. The crystallinity and surface area of the loaded nanoclusters over Co_3_O_4_ were measured by X-ray diffraction (XRD) and N_2_ adsorption/desorption isotherms at − 196 °C. X-ray photoelectron spectroscopy (XPS) confirmed that the charge of Pd and Pt in the monometallic and bimetallic nanoclusters is zero.

## Experimental

### Chemicals

Potassium hexachloropalladate (IV) (K_2_PdCl_6_, 98%, Sigma–Aldrich), chloroplatinic acid (H_2_PtCl_6_.6H_2_O, ≥ 37.5% Pt basis, Sigma–Aldrich), sodium borohydride (NaBH_4_, ≥ 96%, Aldrich), l-glutathione (99%, Sigma–Aldrich) and ethanol were used for preparing the protected Pd and Pt nanoclusters and the Pd–Pt alloy nanoclusters. Cobalt (II, III) oxide (Co_3_O_4_) nanopowder, < 50 nm particle size (99.5%, Sigma–Aldrich), was used as active support. All chemicals were used as received. All glassware was thoroughly cleaned with aqua regia (HCl:HNO_3_ = 3:1 v/v), rinsed with double distilled water and ethanol, and dried in an oven before use.

### Preparation of Pd_n_(SG)_m_ nanoclusters

79.466 mg potassium hexachloropalladate (IV) (K_2_PdCl_6_, 0.2 mmol) was dissolved in 10 mL double-distilled water, 43.02 mg l-glutathione (0.14 mmol) was dissolved in 3 mL double-distilled water, and added to the palladium solution, under vigorously stirring (~ 1100 rpm) at room temperature. During the addition of the l-glutathione solution, the orange color of the palladium salt solution became light orange, and then the color changed to red. After 30 min. of stirring, a freshly prepared aqueous solution of NaBH_4_ (75.66 mg, 2 mmol, dissolved in 3 mL double-distilled water) was added dropwise over the resulting solution while stirring vigorously (~ 1100 rpm). The red solution converted quickly to black, indicating the reduction of the palladium salt and the formation of palladium nanoclusters. The reaction was allowed to proceed under constant stirring for 1 h. The mixture was evaporated under a vacuum to near dryness, and then the particles were precipitated by adding ethanol. The black-brown precipitate was re-dissolved in double-distilled water and re-precipitated by ethanol. This step should be repeated three times to remove excess NaBH_4_. The resulting precipitate was then collected through centrifugal precipitation and washed with ethanol to remove the unreacted material. The black-brown solid consisting of Pd_n_(SG)_m_ nanoclusters was finally dried overnight at 100 °C^26–38^.

### Preparation of Pt_n_(SG)_m_ nanoclusters

81.96 mg chloroplatinic acid (H_2_PtCl_6_.6H_2_O, 0.2 mmol) was dissolved in 10 mL double-distilled water, and 43.02 mg l-glutathione (0.14 mmol) was dissolved in 3 mL double distilled water and added to the platinum solution, under vigorously stirring (~ 1100 rpm) at room temperature. During the addition of the l-glutathione solution, the yellow solution of platinum salt showed some turbidity after adding the ligand, then the color changed to orange and finally to canary yellow. After 30 min stirring, a freshly prepared aqueous solution of NaBH_4_ (75.66 mg, 2 mmol, dissolved in 3 mL double-distilled water) was added dropwise over the resulting solution while stirring vigorously (~ 1100 rpm). The canaries yellow color solution converted gradually to black, indicating the reduction of the platinum salt and formation of platinum nanoclusters. The reaction was allowed to proceed under constant stirring for 1 h. Then, the Pt_n_(SG)_m_ nanoclusters were separated, purified, and dried as described in the case of Pd_n_(SG)_m_ nanoclusters^26^.

### Preparation of (Pd_0.5_–Pt_0.5_)_n_(SG)_m_ nanoclusters

40.98 mg chloroplatinic acid (H_2_PtCl_6_.6H_2_O, 0.1 mmol) was dissolved in 5 mL double-distilled water (solution A). 32.73 mg potassium tetrachloropalladate (II) (K_2_PdCl_4_, 0.1 mmol) was dissolved in 5 mL double-distilled water (solution B). Solution A was mixed with solution B for 30 min., and then 43.02 mg l-glutathione (0.14 mmol) was dissolved in 3 mL double-distilled water and added to the solution vigorously stirring (~ 1100 rpm) at room temperature. The l-glutathione solution reacted with the mixed solutions of the two salts simultaneously. After adding the ligand, the yellow solution showed some turbidity; then the color changed to orange and finally to canary yellow. After 30 min. stirring, a freshly prepared aqueous solution of NaBH_4_ (75.66 mg, 2 mmol, dissolved in 3 mL double-distilled water) was added dropwise over the resulting solution while stirring vigorously (~ 1100 rpm). The canaries yellow color solution converted gradually to black, indicating the platinum and palladium salts reduced simultaneously to form Pd–Pt single alloy nanoclusters. The reaction was allowed to proceed under constant stirring for 1 h. Then, the (Pd_0.5_–Pt_0.5_)_n_(SG)_m_ alloy was separated, purified, and dried as described in the case of Pd_n_(SG)_m_ nanoclusters^26,38^.

### Loading of the prepared nanoclusters over Co_3_O_4_

The 1 wt.% of catalysts (Pd_n_(SG)_m_/Co_3_O_4_, Pt_n_(SG)_m_/Co_3_O_4_, Pd_n_(SG)_m_ + Pt_n_(SG)_m_/Co_3_O_4_ and (Pd_0.5_–Pt_0.5_)_n_(SG)_m_/Co_3_O_4_) were prepared by a well-known impregnation method. 0.5 g of Co_3_O_4_ nanoparticles were suspended in 50 mL double-distilled water in an ultrasonic bath for 30 min., 5 mg of the clusters were dissolved in 10 mL double-distilled water, and then added to the Co_3_O_4_ slurry. The slurry was stirred 24 h at room temperature, and then the loaded catalyst was collected by centrifuge at 6000 rpm, for 10 min. The brown color of the clusters solution becomes colorless, confirming the transfer of all clusters amount to the Co_3_O_4_ surface. The loaded catalysts were dried in an oven at 100 °C overnight^26,38^.

### The catalytic reaction for the hydrolysis of NaBH_4_

The hydrolysis of NaBH_4_ has been performed at room temperature (25 °C), over 5 mg of the prepared catalysts (Pd_n_(SG)_m_/Co_3_O_4_, Pt_n_(SG)_m_/Co_3_O_4_, Pd_n_(SG)_m_ + Pt_n_(SG)_m_/Co_3_O_4_, and (Pd_0.5_–Pt_0.5_)_n_(SG)_m_/Co_3_O_4_). 1 g of NaBH_4_ was added to 100 mL of water (corresponding to 1 wt.%), and the catalyst was added. The reaction solution was stirred at 1000 rpm. The hydrogen-generated volume was measured using the water displacement method^9–12^. (Pd_0.5_–Pt_0.5_)_n_(SG)_m_/Co_3_O_4_ was used as a model catalyst to study the effect of catalyst weight (5, 15, 30, and 50 mg) in the hydrolysis of 1 wt.% NaBH_4_ solution at 25 °C.

5 mg of the (Pd_0.5_–Pt_0.5_)_n_(SG)_m_/Co_3_O_4_ catalyst was chosen to employ different NaBH_4_ solutions such as 0.189, 0.5, 1, and 2 wt.%. The same amount of the catalyst was used to follow up the reaction at different temperatures 25, 45, and 60 °C, of the 1 wt.% NaBH_4_ solution.

In the recyclability study, 5 mg of (Pd_0.5_–Pt_0.5_)_n_(SG)_m_/Co_3_O_4_ catalyst was repeatedly used five times to generate H_2_ using 1 wt.% NaBH_4_ solution at 25 °C. Once the hydrolysis reaction of NaBH_4_ was completed, a new NaBH_4_ solution was added for a second cycle, and so on.

## Results and discussion

### Optical properties and chemical composition of the prepared nanoclusters

Metal nanoclusters exhibit some absorption in a visible region, and these absorptions come from the excitation of plasmon resonance electrons or interband transitions^26,30,31,33,38^. These absorption peaks' positions and width depend on the metal type, particle size, protecting ligand, and chemical charge^26,29,31,33,38^. Pt nanoparticles in regime 4–10 nm show an absorption peak at 215 nm^39,40^. The intensity of this peak decreases by decreasing particle size and is essentially undetectable for smaller clusters less than 2 nm^41^. S-containing Pd(II) complexes exhibited surface plasmon peak at 350–500 nm, corresponding to the charge transfer between the metal and ligand^42^.

**Figure 1:**
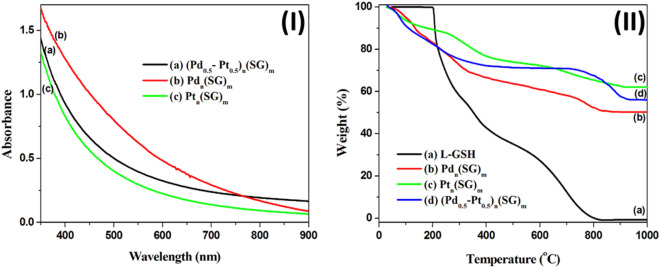
(**I**) UV–Vis absorption spectra of the prepared nanoclusters (Pd_0.5_–Pt_0.5_)_n_(SG)_m_ (a), Pd_n_(SG)_m_ (b), and Pt_n_(SG)_m_ (c). The prepared clusters show featureless absorption curves in the visible region of 350–900 nm. (**II**) The thermogravimetric analysis (TGA) of the prepared clusters ((Pd_0.5_–Pt_0.5_)_n_(SG)_m_ (a), Pd_n_(SG)_m_ (b), and Pt_n_(SG)_m_ (c)) and the protecting ligand l-glutathione (d).

The prepared Pd and Pt nanoclusters and Pd/Pt alloy nanoclusters in water show featureless absorption curves in the UV–vis region, where their color is dark brown (Fig. 1-I). These absorption curves agree with the theoretical calculations by Creighton and Eadon^39^. As shown in Fig. 1-I, there are no peaks or noise in the 350–500 nm region, indicating the prepared nanoclusters are pure from the intermediated complex (Pd(II)-SG), and all the palladium ions are reduced to Pd(0)^33^. Moreover, the prepared nanoclusters are pure from the unreacted l-glutathione ligand, which shows one absorption peak at 224 nm^38^.

Thermogravimetric analysis (TGA) is used to obtain the ratio between the organic and metallic parts in the protected nanoclusters^26,29,31,33,38^. Therefore, the metal-to-ligand ratio (M/L) and the average chemical formula of the MPCs can be estimated. Figure 1-II shows the TGA curves of the prepared nanoclusters and the pure ligand. According to the TGA curves, the protecting ligand in Pd_n_(SG)_m_, Pt_n_(SG)_m,_ and (Pd_0.5_–Pt_0.5_)_n_(SG)_m_ nanoclusters decompose in two decomposition steps. The ligand decomposition is completed at 900 °C; the residual is the metallic part. The weight percentage of the metallic part of the prepared nanoclusters Pd_n_(SG)_m_, Pt_n_(SG)_m_, and (Pd_0.5_–Pt_0.5_)_n_(SG)_m_ are 50.18, 62.1, and 56 wt. % (Table 1). The average metal/ligand ratio (M/L) and the average molecular formula of the prepared nanoclusters are calculated and summarized in Table 1^26,38^.

**Table 1:** The thermogravimetric analysis data for the prepared nanoclusters.

Catalysts	Weight (%) at 900 °C	M/S ratio	Molecular formula
(Pd_0.5_–Pt_0.5_)_n_(SG)_m_	56.00	1:0.38	(Pd–Pt)_3n_L_n_
Pd_n_(SG)_m_	50.18	1:0.34	Pd_3n_L_n_
Pt_n_(SG)_m_	62.10	1:0.30	Pt_3n_L_n_

### The particles size and charge of the prepared nanoclusters

**Figure 2:**
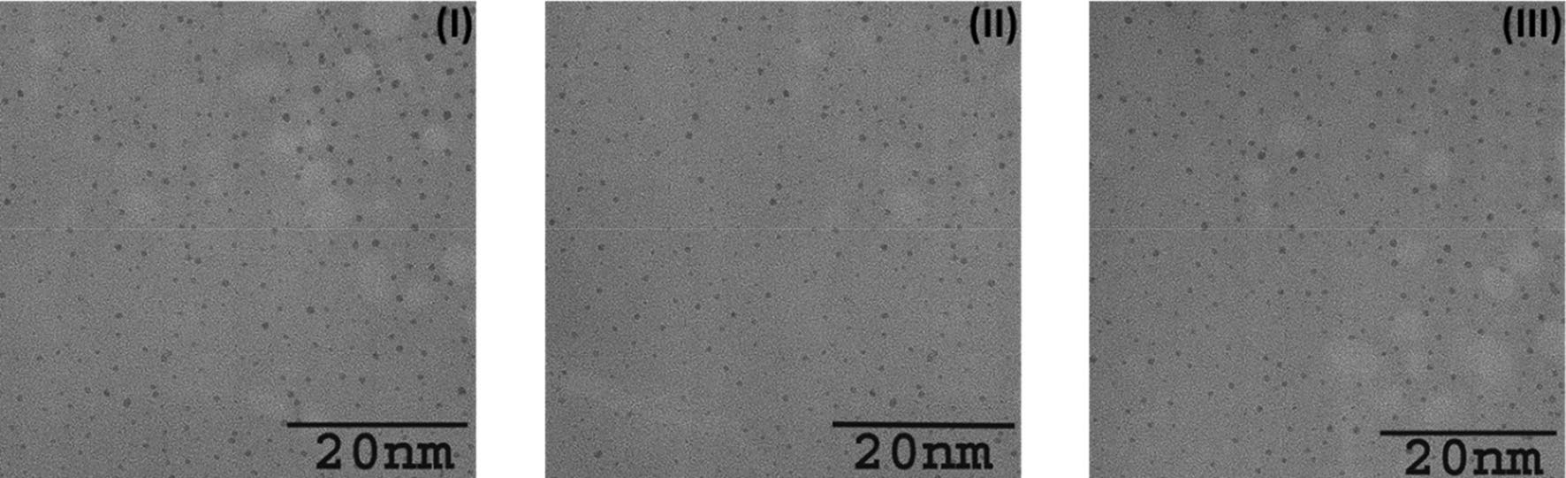
High resolution-transmission electron microscopy (HR-TEM) images of the prepared clusters Pd_n_(SG)_m_ (**I**), Pt_n_(SG)_m_ (**II**), and (Pd_0.5_–Pt_0.5_)_n_(SG)_m_ (**III**). The three clusters show a monodisperse particle size of around 1 nm.

High resolution-transmission electron microscopy (HR-TEM) was used to measure the particle size of the prepared nanoclusters. Figure 2 (I, II, and III) shows the HR-TEM images of the Pd_n_(SG)_m_, Pt_n_(SG)_m_, and (Pd_0.5_–Pt_0.5_)_n_(SG)_m_, respectively. The particle size of the prepared clusters is ~ 1 nm, which appears as QDs. The single alloy QDs (Pd_0.5_–Pt_0.5_)_n_(SG)_m_) shows a particle size that is uniformly distributed similar to the monometallic nanoclusters (Pd_n_(SG)_m_ and Pt_n_(SG)_m_). This indicates that the method can be used for bimetallic and monometallic QDs preparation.

**Figure 3:**
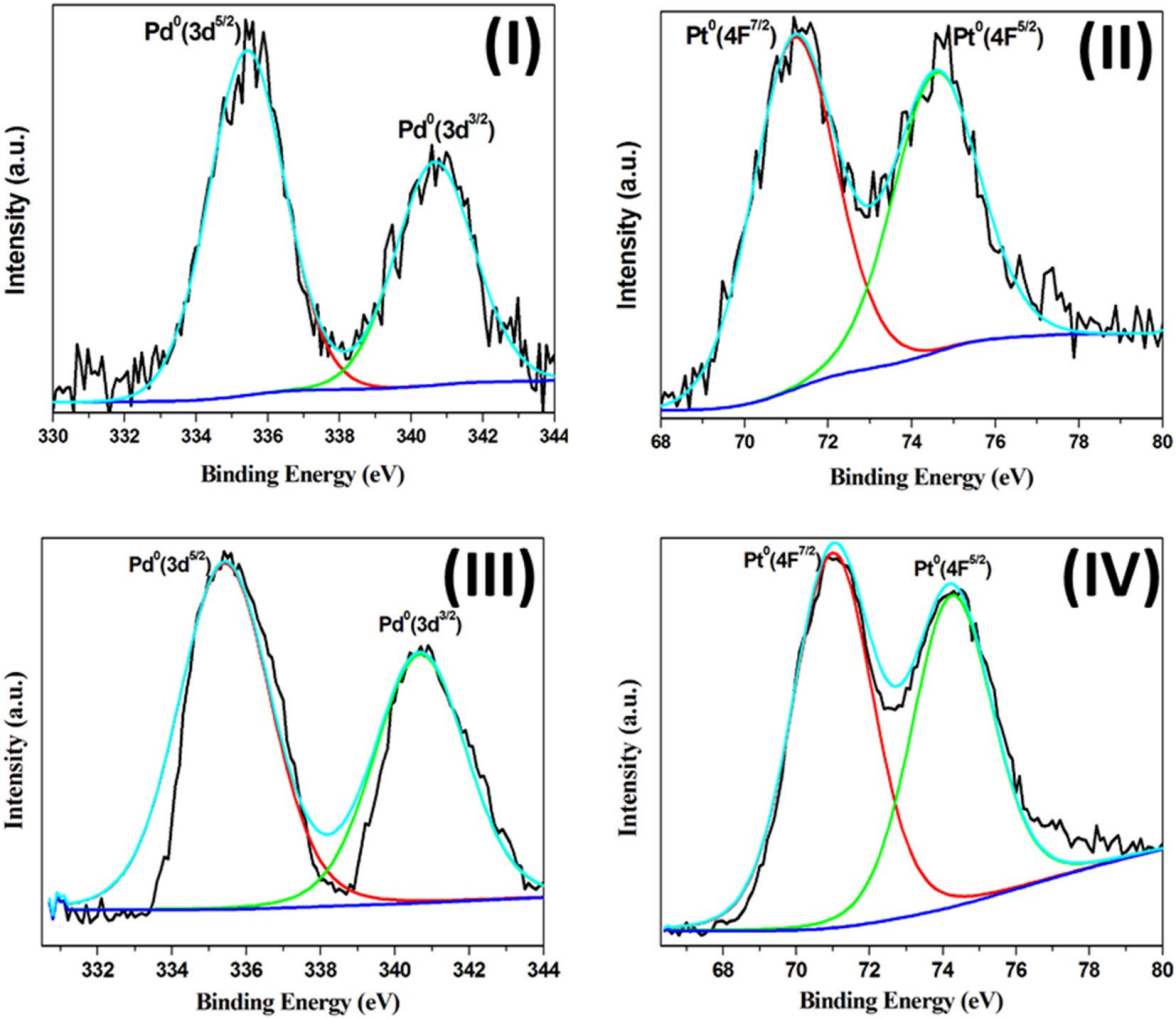
High resolution-XPS spectra of (**I**) Pd 3d^5/2^ and 3d^3/2^ electrons in Pd_n_(SG)_m_ clusters, (**II**) Pt 4F^5/2^ and 4F^7/2^ electrons in Pt_n_(SG)_m_ clusters, (**III**) Pd atoms in the single alloy nanoclusters ((Pd_0.5_–Pt_0.5_)_n_(SG)_m_), and (**IV**) Pt atoms in the single alloy nanoclusters ((Pd_0.5_–Pt_0.5_)_n_(SG)_m_).

The particle charge of the prepared nanoclusters is characterized by X-ray photoelectron spectroscopy (XPS). The Pd 3d XPS spectrum of the Pd_n_(SG)_m_ clusters is shown in Fig. 3-I. The binding energies of the Pd 3d^5/2^ and 3d^3/2^ electrons are at 335.42 and 340.66 eV^16^ respectively, corresponding to Pd(0). These findings are in agreement with those obtained for Pd nanoparticles loaded on Co_3_O_4_ nanoparticles^43,44^. Figure 3-II shows the XPS spectrum of platinum atoms in Pt_n_(SG)_m_ clusters, the binding energies of the Pt 4F^5/2^ and 4F^7/2^ electrons are 74.58 and 71.19 eV, respectively, that are corresponding to Pt(0)^45^. The charge of Pd and Pt atoms in the single alloy nanoclusters (Pd_0.5_–Pt_0.5_)_n_(SG)_m_ are also zero, as confirmed by XPS analysis (Fig. 3-III,IV), where the binding energies of the Pd 3d^5/2^ and 3d^3/2^ electrons are 335.44 and 340.70 eV, and for Pt 4F^5/2^ and 4F^7/2^ electrons are 74.36 and 71.02 eV, respectively. The absence of the two peaks of Pd^4+^ at 339.2 and 342.9 eV indicates the presence of Pd in metallic form (neither as PdO_2_ nor Pd(OH)_4_)^43^.

### Texture properties and crystallinity of the prepared catalysts

**Figure 4:**
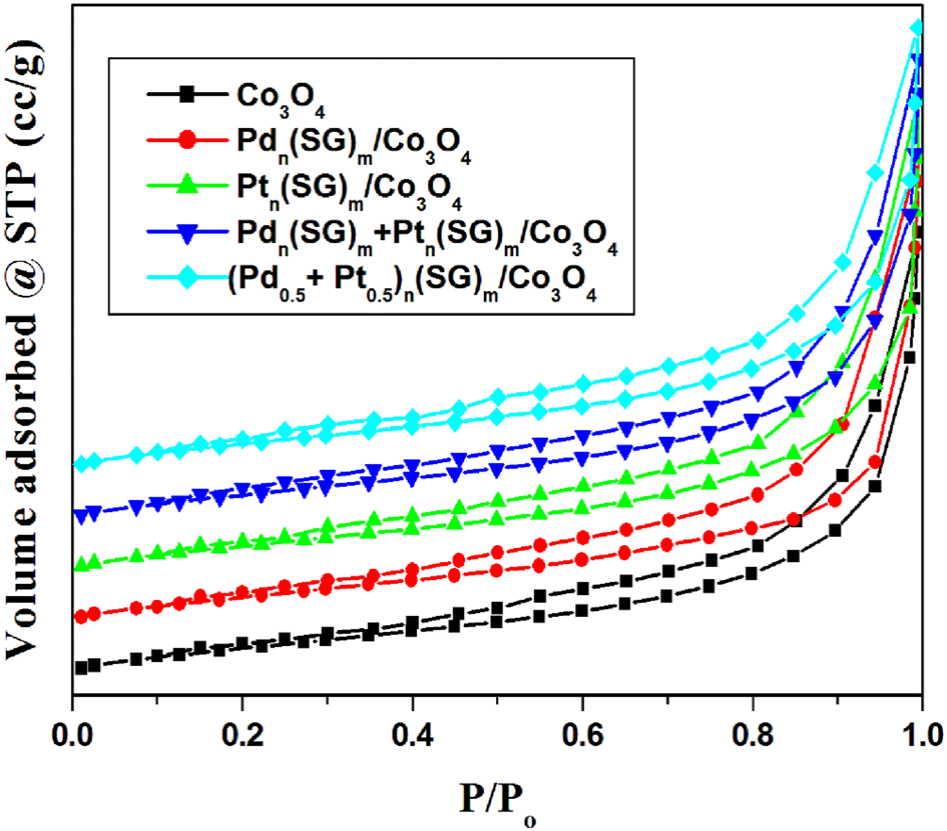
N_2_ adsorption/desorption isotherms of the prepared catalysts and pure support show sorption isotherms type H3 according to the IUPAC classification.

Liquid nitrogen at − 196 °C is used to determine the surface texture properties of the pure support (Co_3_O_4_) and the doped Co_3_O_4_ with the prepared nanoclusters (Pd_n_(SG)_m_/Co_3_O_4_, Pt_n_(SG)_m_/Co_3_O_4_, Pd_n_(SG)_m_ + Pt_n_(SG)_m_/Co_3_O_4_ and (Pd_0.5_–Pt_0.5_)_n_(SG)_m_/Co_3_O_4_) by measuring the adsorption/desorption isotherms (Fig. 4). According to the IUPAC classification of hysteresis loops, the sorption isotherms of the prepared catalysts and the pure support are type H3 hysteresis loops^46–48^. The surface areas of the prepared catalysts and support are summarized in Table 2. The values of surface area that are measured by BET equation (S_BET_) and T-method (S_t_) for all the investigated catalysts are close to each other, which confirms the correct choice of standard t-curves for pore analysis and indicates the absence of ultra-micropores in these catalysts^26,33^.

**Table 2:** Specific surface area data for the prepared catalysts and pure support**.**

Catalysts	S_BET_ (m^2^ g^− 1^)	S_t_ (m^2^ g^− 1^)
(Pd_0.5_–Pt_0.5_)_n_(SG)_m_/Co_3_O_4_	56.3	56.3
Pd_n_(SG)_m_ + Pt_n_(SG)_m_/Co_3_O_4_	54.7	54.7
Pd_n_(SG)_m_/Co_3_O_4_	56.8	56.8
Pt_n_(SG)_m_/Co_3_O_4_	55.2	55.2
Co_3_O_4_	60.3	60.3

**Figure 5:**
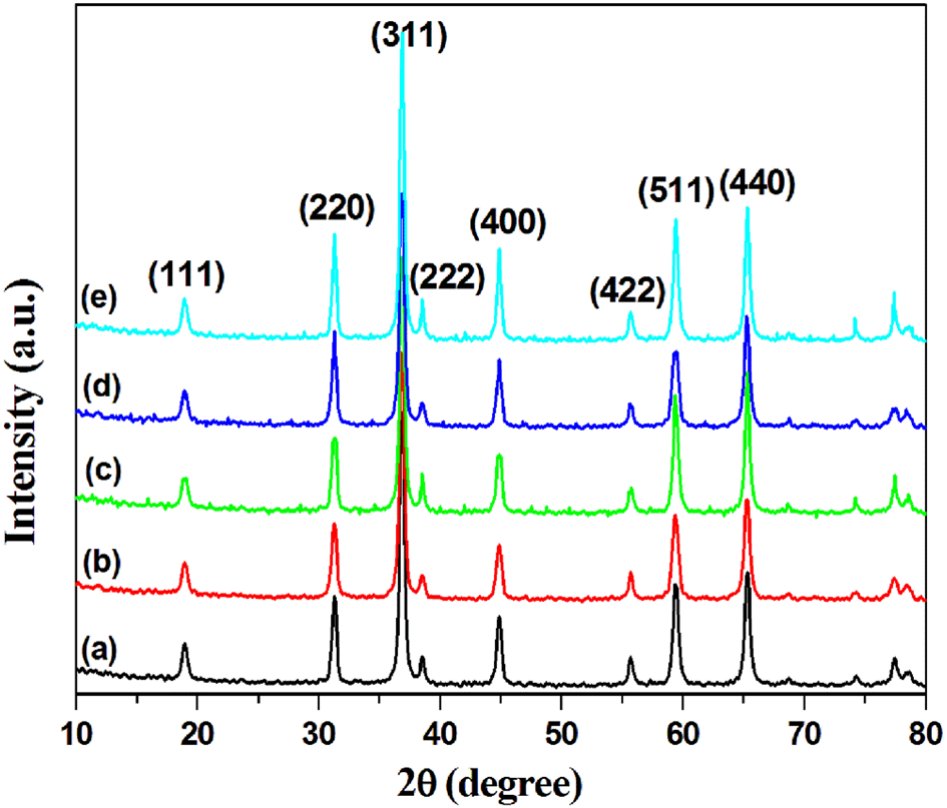
X-ray diffractograms of the pure Co_3_O_4_ support (a), and prepared catalysts: Pd_n_(SG)_m_/Co_3_O_4_ (b), Pt_n_(SG)_m_/Co_3_O_4_ (c), Pd_n_(SG)_m_ + Pt_n_(SG)_m_/Co_3_O_4_ (d), and Pd_0.5_–Pt_0.5_)_n_(SG)_m_/Co_3_O_4_ (e).

X-ray diffraction is used to measure the crystallinity of the prepared catalysts. Figure 5 illustrates the XRD diffractograms of the prepared catalysts and Co_3_O_4_. The characteristic diffraction planes of the Co_3_O_4_ are (111), (220), (311), (222), (400), (422), (511) and (440) appear at 2θ equal to 19.0°, 31.2°, 36.7°, 38.5°, 44.8°, 55.5°, 59.3° and 65.4°, respectively (Fig. 5a)^49,50^. The prepared catalysts show the same XRD pattern as pure Co_3_O_4,_ and no new peaks related to the presence of Pd or Pt clusters are detected, where the doping percentage is very low (1 wt.%) (Fig. 5b–e). This means the crystallinity of pure Co_3_O_4_ does not affect loading with the prepared clusters. Also, there is no significant difference in either diffraction angle or peak width between the Co_3_O_4_ and prepared catalysts, indicating that the crystal structure of Co_3_O_4_ is not affected by doping. The crystalline size (d_Co3O4_) of bare Co_3_O_4_ is calculated using Debye–Scherrer Equations^51^ as 20 nm for the broadening of (311) peak reflection (Fig. 5). The d_Co3O4_ value for 1 wt.% (Pd_0.5_–Pt_0.5_)_n_(SG)_m_/Co_3_O_4_ catalyst is less than bare Co_3_O_4_ 18.6 nm (Fig. 5)^26,33^.

### The catalytic activity of the prepared catalysts

**Figure 6:**
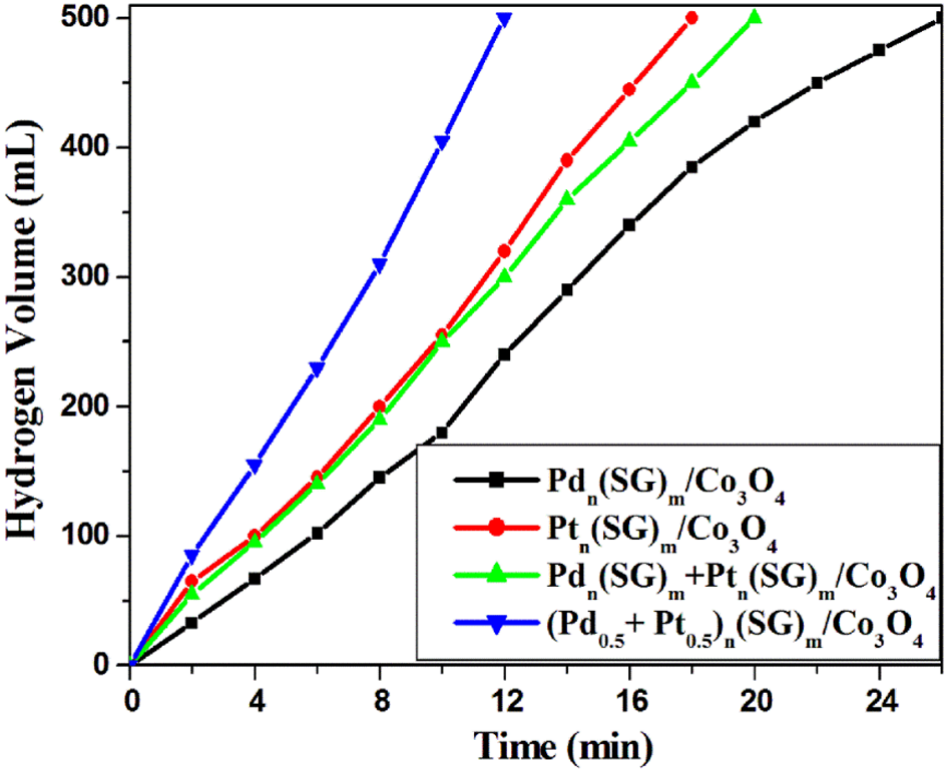
Hydrogen generation from NaBH_4_ hydrolysis over the prepared catalysts. Reaction conditions: 1 g of NaBH_4_ in 100 H_2_O; catalyst, 5 mg; at 25 °C.

The hydrolysis reaction of NaBH_4_ for hydrogen generation is chosen as a model reaction to estimate the catalytic activities of Pd_n_, Pt_n_, physical mixture (Pd_n_ + Pt_n_) clusters and the single alloy clusters (Pd_0.5_–Pt_0.5_)_n_ over Co_3_O_4_ nanoparticles as support. Figure 6 shows the volume of generated hydrogen at room temperature (25 °C) against reaction time over the prepared catalysts. 5 mg of 1 wt.% Pd_n_(SG)_m_/Co_3_O_4_ catalyst produces the maximum hydrogen volume i.e. 500 mL from the hydrolysis of NaBH_4_ solution (1 g NaBH_4_ in 100 mL H_2_O) within 26 min. stirring. Pt_n_(SG)_m_ nanoclusters over Co_3_O_4_ show more activity than Pd clusters in the hydrolysis of NaBH_4_ solution, where the Pt_n_(SG)_m_/Co_3_O_4_ catalyst produces the maximum hydrogen volume within 18 min. stirring at room temperature (Fig. 6). However, the physical mixing of Pd and Pt clusters over Co_3_O_4_ with the same doping percentage (~ 1 wt.%) shows lower catalytic activity than 1 wt. % Pt_n_(SG)_m_/Co_3_O_4_ catalyst. Surprisingly, the single alloy of Pd and Pt QDs (Pd_0.5_–Pt_0.5_)_n_(SG)_m_/Co_3_O_4_) shows amazing catalytic activity, where it reaches the maximum hydrogen volume i.e. 500 mL, within only 12 min. stirring (Fig. 6) with HGR of 8333 mL min^− 1^ g^− 1^. The HGRs for the NaBH_4_ hydrolysis reaction over the prepared catalysts are summarized in Table 3.

**Table 3:** Hydrogen generation rate (HGR) for NaBH_4_ hydrolysis reaction over different catalysts.

Catalysts	HGR (mL min^− 1^ g^− 1^)
(Pd_0.5_–Pt_0.5_)_n_(SG)_m_/Co_3_O_4_	8333
Pd_n_(SG)_m_ + Pt_n_(SG)_m_/Co_3_O_4_	5000
Pt_n_(SG)_m_/Co_3_O_4_	5555
Pd_n_(SG)_m_/Co_3_O_4_	3846

Catalytic test: 1 g of NaBH_4_ in 100 H_2_O; Catalyst, 5 mg; at 25 °C.

The excellent synergetic interaction between Pd and Pt atoms in the single alloy (Pd_0.5_–Pt_0.5_)_n_(SG)_m_ QDs plays a crucial role in the observed high catalytic activity. This positive synergistic effect does not appear in the physical mixing of nanoclusters over the support (Pd_n_(SG)_m_ + Pt_n_(SG)_m_/Co_3_O_4_) by the same doping percentage. Since there are two types of synergetic effect (positive and negative)^52^, if the qualitative effect of electronic interaction between the components of the bimetallic system and the geometric effects due to changes in lattice constants are in the same direction as the bimetallic system will enhance the reaction effectively in comparison to the monometallic systems. However, if the electronic and geometric effect direction is opposite, the lower activity will be received^52^.

**Figure 7:**
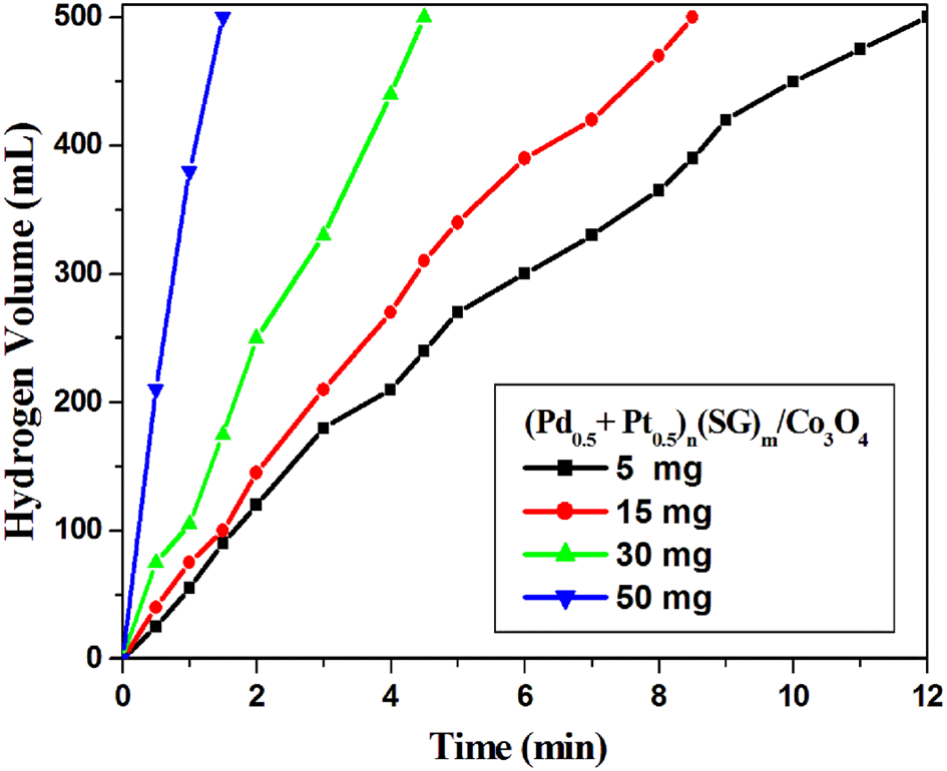
Effect of catalyst weight for hydrogen generation from the hydrolysis of NaBH_4_ over 1 wt.% (Pd_0.5_–Pt_0.5_)_n_(SG)_m_/Co_3_O_4_. Reaction conditions: 1 g of NaBH_4_ in 100 H_2_O; catalyst, 5–50 mg; at 25 °C.

Figure 7 shows the effect of catalyst weight on the hydrolysis of NaBH_4_. (Pd_0.5_–Pt_0.5_)_n_(SG)_m_/Co_3_O_4_ catalyst was chosen to study this factor. 5, 15, 30, and 50 mg of the catalyst were added to the NaBH_4_ solution (1 g NaBH_4_ in 100 mL H_2_O) at room temperature (25 °C). As shown in Fig. 7, the time to reach the maximum hydrogen volume (500 mL) decreases with the increase in the weight of the catalyst. Where the time to reach the maximum hydrogen volume decreases from 12.0 to 1.5 min. over 5 and 50 mg of (Pd_0.5_–Pt_0.5_)_n_(SG)_m_/Co_3_O_4_, respectively (Fig. 7).

**Figure 8:**
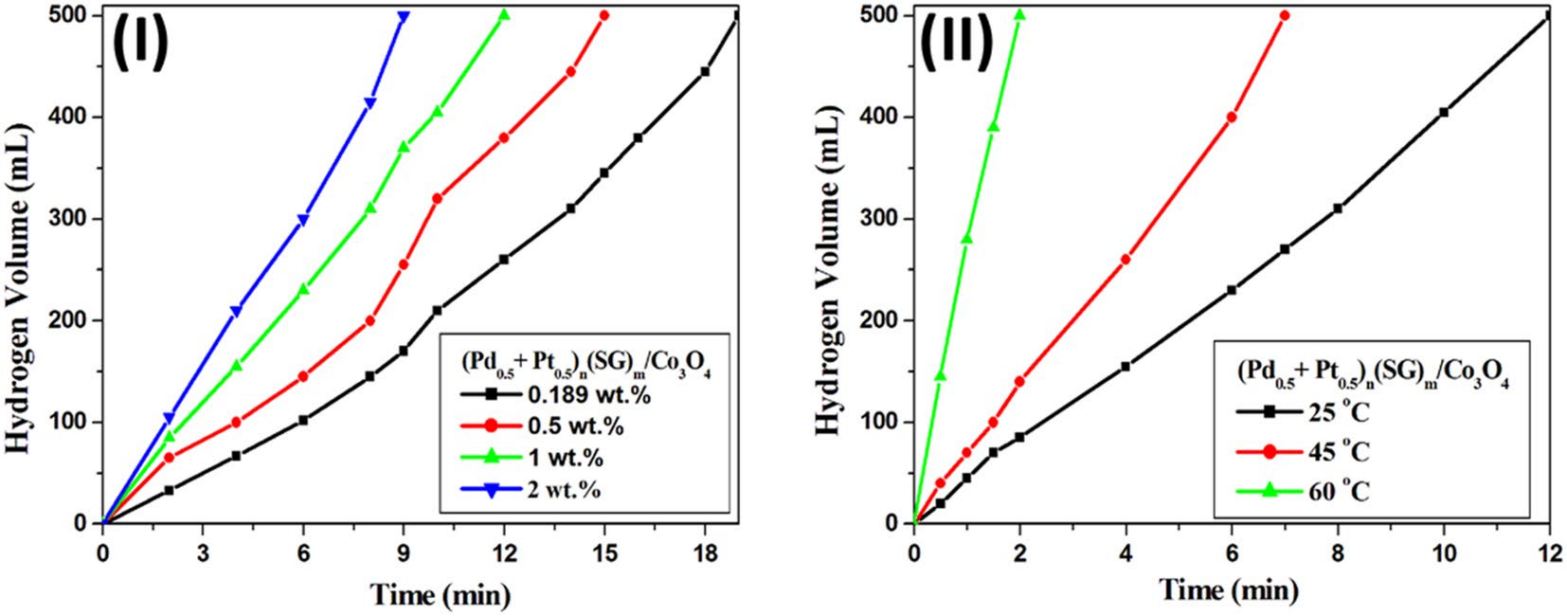
(**I**) Effect of NaBH_4_ amount for hydrogen generation over 1 wt.% (Pd_0.5_–Pt_0.5_)_n_(SG)_m_/Co_3_O_4_. Reaction conditions: 0.189, 0.5, 1 and 2 g NaBH_4_ amount in 100 H_2_O; Catalyst, 5 mg; at 25 °C. (**II**) Effect of reaction temperature for hydrogen generation from the hydrolysis of NaBH_4_ over 1 wt.% (Pd_0.5_–Pt_0.5_)_n_(SG)_m_/Co_3_O_4_. Reaction conditions: 1 g of NaBH_4_ in 100 H_2_O; catalyst, 5 mg; at 25–60 °C.

Different concentrations of NaBH_4_ solution (0.189, 0.5, 1 and 2 wt.%) were tested over 5 mg of (Pd_0.5_–Pt_0.5_)_n_(SG)_m_/Co_3_O_4_ catalyst at 25 °C (Fig. 8-I). The reaction time decreases with the increase of the NaBH_4_ concentration. The maximum hydrogen volume was reached within 19, 15, 12, and 9 min. of the 0.189, 0.5, 1, and 2 wt.% NaBH_4_ solution over (Pd_0.5_–Pt_0.5_)_n_(SG)_m_/Co_3_O_4_ catalyst, respectively (Fig. 8-I). The effect of reaction temperature was also tested for hydrogen generation from the 1 wt.% NaBH_4_ solution over 5 mg of (Pd_0.5_–Pt_0.5_)_n_(SG)_m_/Co_3_O_4_ catalyst (Fig. 8-II). The reaction was measured at different temperatures at 25, 45, and 60 °C; the reaction time decreased with the temperature increasing (Fig. 8-II). The maximum hydrogen volume was reached within only 2 min stirring over 5 mg of (Pd_0.5_–Pt_0.5_)_n_(SG)_m_/Co_3_O_4_ catalyst at 60 °C with HGR 50,000 mL min^− 1^ g^− 1^ (Fig. 8-II).

**Figure 9:**
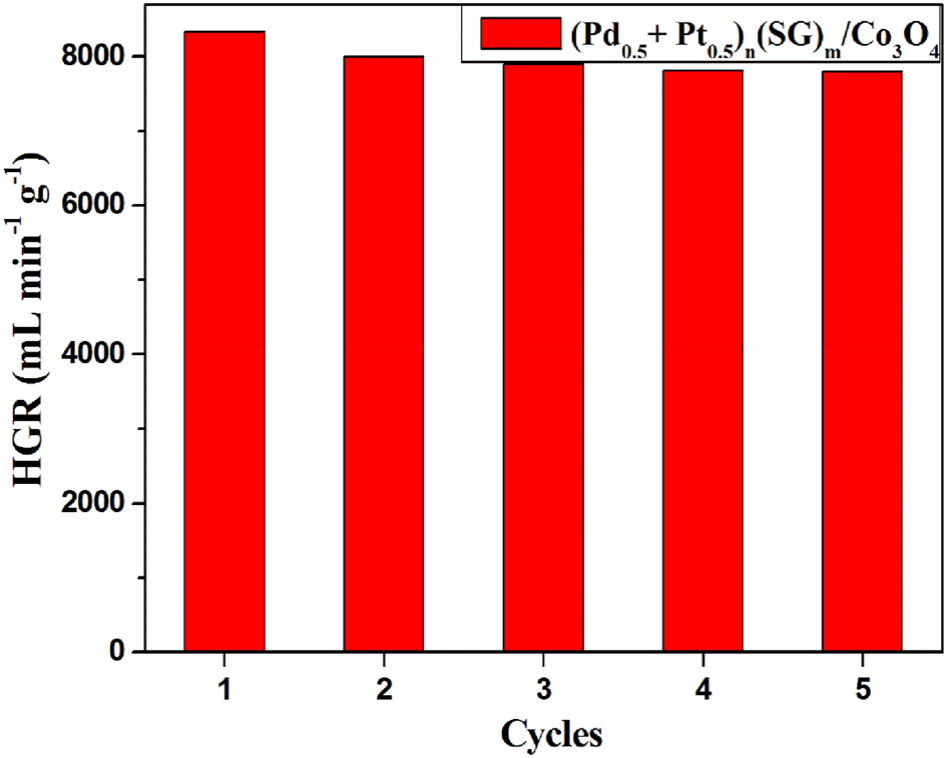
Recyclability of 1 wt.% (Pd_0.5_–Pt_0.5_)_n_(SG)_m_/Co_3_O_4_ catalyst for hydrogen generation from the hydrolysis of NaBH_4_. Reaction conditions: 1 g of NaBH_4_ in 100 H_2_O; catalyst, 5 mg; at 25 °C.

The best property of heterogeneous catalysis is easily separating the catalyst from the reaction mixture and reusing it until its activity decreases^26–28,32,33,38^. Thus, the ability of the (Pd_0.5_–Pt_0.5_)_n_(SG)_m_/Co_3_O_4_ catalyst to recycle is tested for the hydrolysis of 1 wt. % NaBH_4_ solution at room temperature using 5 mg from the catalyst. After each run, the catalyst was collected and reused without any treatment. The HGRs values were nearly identical for the 5 cycles (Fig. 9). These results reveal that the (Pd_0.5_–Pt_0.5_)_n_(SG)_m_/Co_3_O_4_ catalyst can be reused, and their catalytic activity is quite consistent without significant change.

**Figure 10:**
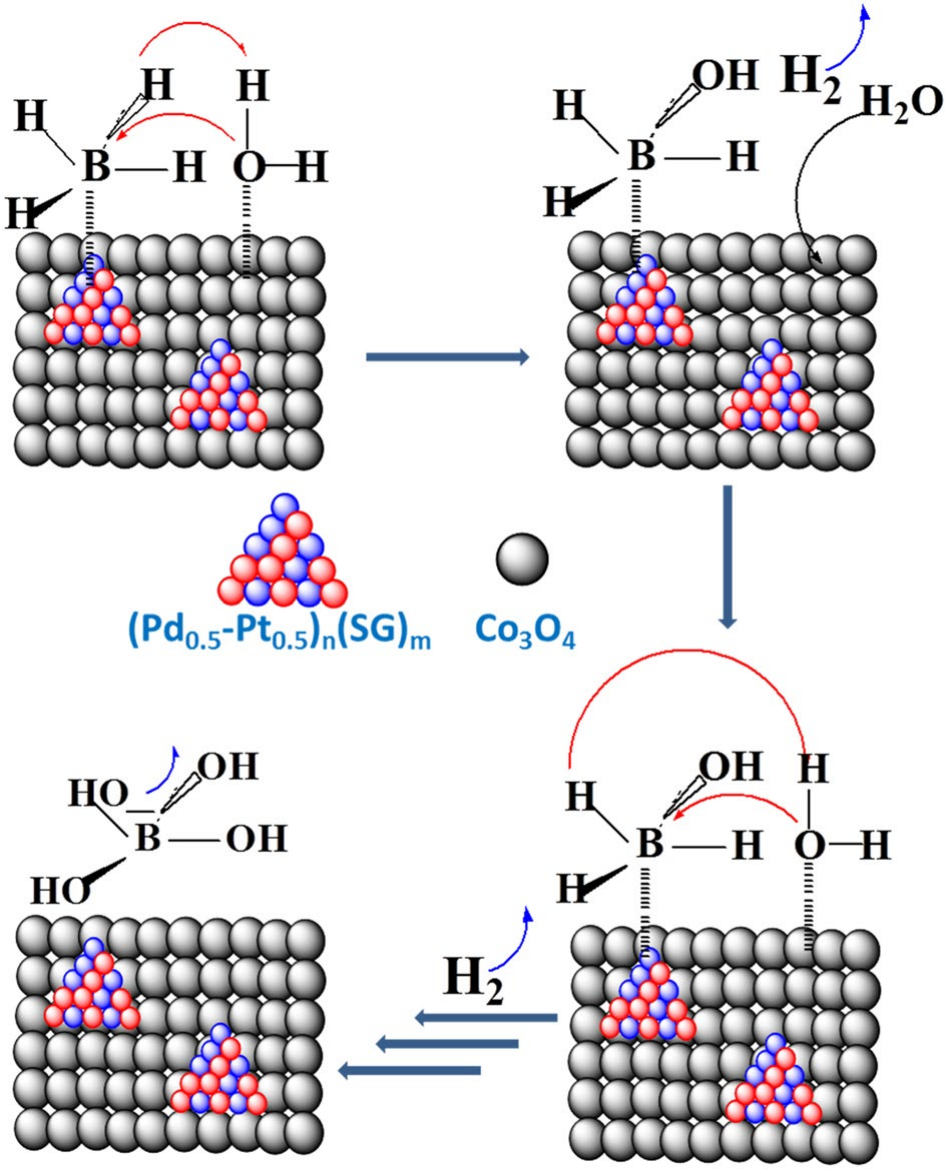
Suggested mechanism of NaBH_4_ hydrolysis over (Pd_0.5_–Pt_0.5_)_n_(SG)_m_/Co_3_O_4_ catalyst, according to the Langmuir–Hinshelwood model.

The NaBH_4_ hydrolysis reaction mechanism can be further illustrated in view of Langmuir–Hinshelwood model. Where, Kojima et al.^53^ reported the hydrolysis mechanism of NaBH_4_ over Pt/LiCoO_2_ catalyst, according to the Langmuir–Hinshelwood model. It was found that BH_4_^−^ ions are adsorbed on Pt, while H_2_O molecules are adsorbed on the oxide support to give H_2_ and B(OH)_4_^−^^53^. These findings were confirmed by Liu et al.^54^ using X-ray absorption. Co_3_O_4_ played the same role as LiCoO_2_ for Pt/Co_3_O_4_ catalyst as claimed by Hung et al.^55^. The suggested mechanism of NaBH_4_ hydrolysis over the (Pd_0.5_–Pt_0.5_)_n_(SG)_m_/Co_3_O_4_ catalyst is demonstrated in Fig. 10, according to the Langmuir–Hinshelwood model. The catalytic process involves two different adsorption sites. The first site is suggested to be electron-rich Pt^0^ and Pd^0^ nanoclusters, whereas the second site is electron-deficient (Co^δ+^). The hydrolysis of the four hydrides of BH_4_^−^ occurs one after the other by reaction with one adsorbed H_2_O for each hydride (Fig. 10)^56^.

A comparison with different catalysts used for hydrogen generation via NaBH_4_ hydrolysis is listed in Table 4. As shown in Table 4, the prepared catalysts in this work showed superior catalytic activity in hydrogen generation from the hydrolysis of NaBH_4_ using a relatively low noble metal loading (1 wt.%). Where (Pd_0.5_–Pt_0.5_)_n_(SG)_m_/Co_3_O_4_ catalyst showed the highest hydrogen yield in the Table 4 with HGR equal to 8333 mL g^−1^ min^−1^. Moreover, 1 wt.% loading percentage of monometallic Pt and Pd clusters over Co_3_O_4_ exhibited HGR 5555 and 3846 mL g^−1^ min^−1^, however, the 20 wt.% Pt and Pd nanoparticles over the same support exhibited only 4713 and 2109 mL g^−1^ min^−1^^11^. This high catalytic activity is due to the unique atomic packing structure and electronic properties of the nanoclusters.

**Table 4:** Comparison among catalysts used for the hydrolysis of NaBH_4_ for hydrogen generation.

Catalysts	Catalyst mass (mg)	NaBH_4_ (wt.%)	HGR (mL min^−1^ g^−1^)	Ref
5.4 wt.% Ru/Al_2_O_3_	500	10	65.5	^57^
Pt–Pd/CNT	–	0.1	126	^58^
10 wt.% PtRu/LiCoO_2_	125	5	30	^20^
2 wt.% Ru/MMT	32	1	541	^59^
5 wt.%CoB/CeO_2_	200	1	533	^10^
3 wt.% Ru/graphite	300	10	666.6	^60^
20 wt.% Pd/Co_3_O_4_	50	10	2109	^11^
Pt/LiCoO_2_	50	20	3100	^53^
20 wt.% Pt/Co_3_O_4_	50	10	4713	^11^
1 wt.%(Pd_0.5_–Pt_0.5_)_n_(SG)_m_/Co_3_O_4_	5	1	8333	This Work
1 wt.% Pt_n_(SG)_m_/Co_3_O_4_			5555	
1 wt.% Pd_n_(SG)_m_/Co_3_O_4_			3846	

## Conclusions

In conclusion, a single alloy of Pd/Pt quantum dots over Co_3_O_4_ (Pd_0.5_–Pt_0.5_)_n_(SG)_m_/Co_3_O_4_) exhibited promising catalytic activity in hydrogen generation from the hydrolysis of NaBH_4_. The maximum hydrogen volume i.e. 500 mL was reached within only 12 min. of stirring at room temperature with HGR 8333 mL min^− 1^ g^− 1^. However, the physical mixing of the prepared two clusters (Pd_n_(SG)_m_ and Pt_n_(SG)_m_) over Co_3_O_4_ exhibited a lower hydrogen yield in comparison to the single alloy clusters. The time to reach the maximum hydrogen volume decreases with the increase of the (Pd_0.5_–Pt_0.5_)_n_(SG)_m_/Co_3_O_4_ catalyst weight (5, 15, 30, and 50 mg). Moreover, the effect of NaBH_4_ concentration (0.189, 0.5, 1 and 2 wt.%) over 5 mg of (Pd_0.5_–Pt_0.5_)_n_(SG)_m_/Co_3_O_4_ catalyst at 25 °C was studied. The hydrolysis of the NaBH_4_ reaction was measured at different temperatures of 25, 45, and 60 °C, where 5 mg of (Pd_0.5_–Pt_0.5_)_n_(SG)_m_/Co_3_O_4_ catalyst produced the maximum hydrogen volume within only 2 min stirring at 60 °C, with HGR 50,000 mL min^− 1^ g^− 1^. The extremely catalytic activity of this catalyst attributes to the ultra-small particle size and the synergistic effect between Pd and Pt atoms in the alloy quantum dots.

## Supplementary Information

Supplementary Information.

## Data Availability

All data generated or analyzed during this study are included in this published article (and its Supplementary Information files).
