# Supplementary material for: Hydrogen generation of single alloy Pd/Pt quantum dots over Co3O4 nanoparticles via the hydrolysis of sodium borohydride at room temperature
Source: Sci Rep. 2022 Oct 11;12:17040. doi: 10.1038/s41598-022-21064-z (PMC9553983; doi:10.1038/s41598-022-21064-z)
Supplement: Supplementary file 1 — Supplementary Information. [file 41598_2022_21064_MOESM1_ESM.docx]

**Supplementary Information**

**Hydrogen generation of single alloy Pd/Pt quantum dots over Co_3_O_4_ nanoparticles via the hydrolysis of sodium borohydride at room temperature**

**Mostafa Farrag ^1,*^, Gomaa A.M. Ali ^2^**

^1^ Nanoclusters and Photocatalysis Laboratory, Chemistry Department, Faculty of Science,

Assiut University, 71516 Assiut, Egypt

^2^ Chemistry Department, Faculty of Science, Al-Azhar University, 71524, Assiut, Egypt

* Corresponding author: [mostafafarrag@aun.edu.eg](mailto:mostafafarrag@aun.edu.eg)

**Instrumentation and characterization**

For the UV−Vis absorption spectra of the synthesized nanoclusters, aqueous solutions of approximately 1-2 mg/mL were used. The spectra of all the solutions were recorded at ambient temperature from 350 to 900 nm with a double-beam spectrophotometer (Evolution 300). Thermal gravimetric analysis (TGA, ~ 2 mg) was conducted in an N_2_ atmosphere (flow rate ~50 mL/min) with a Thermo StartTM TG/DAT (Pfeiffer Vacuum). All measurements were performed with a heating rate of 10°C/min, starting from room temperature and ramping up to 1000°C. For TEM measurements, solutions with a concentration of 1−2 mg/mL were prepared by dissolving the samples in double distilled water. A droplet of these samples solutions was casted onto carbon-coated copper grids. The solvent was then allowed to evaporate slowly. TEM images were obtained with a high resolution-transmission electron microscope (HR-TEM) JEOL JEM-2100, JAPAN at an acceleration voltage of 200 kV. The images were then analyzed by using Image J software (version 1.44). Each spectrum was obtained by accumulating 200 scans. Adsorption–desorption isotherms of nitrogen (at −196 ^◦^C) were obtained using a Quantachrome (Nova 3200 series) multi-gas adsorption apparatus. Before analysis, the samples were outgassed at 120 ^o^C for 3 hrs. Specific surface areas were calculated from these isotherms by applying the BET equation. S_t_ Values were calculated using the V_a–t_ plots of de Bore. Powder X-ray diffraction (XRD) was performed on a Philips X-ray powder diffractometer, model PW 2013/00. Ni-filtered Cu Kα with a wavelength of λ = 1.541838 Å was used as a constant source of radiation. The generator was operated at 35 kV and 20 mA, and diffractometer at 50 diverting and receiving slits and a scan rate of 20 mm/min. Fine powder samples were loaded on a quartz plate holder by spreading the powders as a smooth thin layer on the plate. For all diffractograms, the following settings were used: scan range 4–80^◦^ (2θ), scan step 0.06^◦^. The surface electronic states were investigated by using XPS K-ALPHA (Thermo Fisher Scientific) with monochromatic X-ray Al K-alpha radiation -10 to 1350 e.v spot size 400 micro meter at pressure 10^-9^ mbar with full spectrum pass energy 200 e.v and at narrow spectrum 50 e.v. The XPS data were calibrated internally by fixing the BE of the C1s peak at 284.6 eV.
